# Profiling of cell stress protein expression in cardiac tissue of cardiosurgical patients undergoing remote ischemic preconditioning: implications for thioredoxin in cardioprotection

**DOI:** 10.1186/s12967-015-0403-6

**Published:** 2015-01-27

**Authors:** Karina Zitta, Patrick Meybohm, Matthias Gruenewald, Jochen Cremer, Kai D Zacharowski, Jens Scholz, Markus Steinfath, Martin Albrecht

**Affiliations:** Department of Anaesthesiology and Intensive Care Medicine, University Hospital Schleswig-Holstein, Schwanenweg 21, 24105 Kiel, Germany; Department of Anaesthesiology, Intensive Care Medicine and Pain Therapy, University Hospital Frankfurt, Frankfurt am Main, Germany; Department of Cardiovascular Surgery, University Hospital Schleswig-Holstein, Kiel, Germany

**Keywords:** Remote ischemic preconditioning, Cardioprotection, Cardiac surgery, Protein expression, Thioredoxin

## Abstract

**Background:**

Transient episodes of ischemia in a remote organ (remote ischemic preconditioning, RIPC) can attenuate myocardial ischemia/reperfusion injury but the underlying mechanisms of RIPC in the target organ are still poorly understood. Recent animal studies suggested that the small redox protein thioredoxin may be a potential candidate for preconditioning-induced organprotection. Here we employed a human proteome profiler array to investigate the RIPC regulated expression of cell stress proteins and particularly of thioredoxin in heart tissue of cardiosurgical patients with cardiopulmonary bypass (CPB).

**Methods:**

RIPC was induced by four 5 minute cycles of transient upper limb ischemia/reperfusion using a blood pressure cuff. Right atrial tissue was obtained from patients receiving RIPC (N = 19) and control patients (N = 19) before and after CPB. Cell stress proteome profiler arrays as well as Westernblotting and ELISA experiments for thioredoxin (Thio-1) were performed employing the respective tissue samples.

**Results:**

Protein arrays revealed an up-regulation of 26.9% (7/26; CA IX, Cyt C, HSP-60, HSP-70, pJNK, SOD2, Thio-1) of cell stress associated proteins in RIPC tissue obtained before CPB, while 3.8% (1/26; SIRT2) of the proteins were down-regulated. Array results for thioredoxin were verified by semi-quantitative Westernblotting studies which showed a significant up-regulation of thioredoxin protein levels in cardiac tissue samples of RIPC patients taken before CPB (RIPC: 5.36 ± 0.85 a.u.; control: 3.23 ± 0.39 a.u.; P < 0.05). Quantification of thioredoxin levels in tissue of RIPC and control patients by ELISA experiments further confirmed the Westernblotting results (RIPC: 0.30 ± 0.02 ng/mg protein; control: 0.24 ± 0.02 ng/mg protein; P < 0.05).

**Conclusion:**

We provide evidence for thioredoxin as a RIPC-induced factor in heart tissue of cardiosurgical patients and identified several cell stress associated proteins that are regulated by RIPC and may play a role in RIPC-mediated cardioprotection.

**Electronic supplementary material:**

The online version of this article (doi:10.1186/s12967-015-0403-6) contains supplementary material, which is available to authorized users.

## Background

Ischemia/reperfusion injury is commonly found during cardiac surgery and can lead to myocardial and neurological dysfunction as well as increased mortality [1-3].

In the last decades it has been shown that transient episodes of ischemia (ischemic preconditioning) if applied before prolonged ischemia/reperfusion injury have the potential to reduce myocardial infarction [4-9]. Subsequent trials revealed that ischemic preconditioning does not only act locally, but is also able to protect remote tissues from ischemia/reperfusion injury, a phenomenon described as remote ischemic preconditioning (RIPC). RIPC can be induced by inflation and deflation of a blood pressure cuff located at the upper or lower limb. This procedure has been shown to attenuate myocardial injury in a number of clinical situations, especially during cardiac surgery [6,10-16].

RIPC-mediated signalling events within the myocardial tissue possibly involve protein kinase C activation, phosphorylation of pro-survival kinases akt and erk1/2 [17-22] and phosphorylation of STAT5 [23]. Despite the advances in the clinical application of RIPC and increasing knowledge concerning the RIPC-released humoral factors and induced signalling events, there is still an apparent lack of information regarding the proteins that are regulated by RIPC within the myocardial tissue [24,25]. We have recently shown that the activities of matrix metalloproteinases 2 and 9 (MMP-2/9) as well as the expression levels of procaspase-3 and enzymatic activities of myeloperoxidase are increased in heart tissue of cardiosurgical patients receiving RIPC [26,27]. However, the precise cellular mechanisms that are responsible for the RIPC-mediated cardioprotection are still unclear and discussed controversially.

The thioredoxin system is one of the central antioxidant systems in mammalian cells, maintaining a reducing environment by catalyzing electron flux from nicotinamide adenine dinucleotide phosphate through thioredoxin reductase to thioredoxin, which reduces its target proteins using highly conserved thiol groups [28-31]. Interestingly, several authors have suggested that thioredoxin could also be involved in preconditioning induced cardioprotection [32-34].

Here we performed proteomic profiling with cardiac tissue obtained from cardiosurgical patients subjected to RIPC or sham intervention and investigated the expression levels of 26 cell stress associated proteins. We moreover focused on the potential role of thioredoxin by verifying the results of the proteome profiling approach using Westernblotting and ELISA experiments.

## Methods

### Experimental protocol

The study protocol, patient information, and informed consent were approved by the Ethics Committee of the University Hospital Schleswig-Holstein, Campus Kiel, Germany (Reference number: A165/08). The study was performed in accordance with the fourth revision of the Declaration of Helsinki (1996) and is registered at ClinicalTrials.gov (NCT00877305). Employing patient sera and biopsy material, two experimental sub-studies have been published recently ([26,27]; Additional file 1: Table S1).

**Figure 1:**
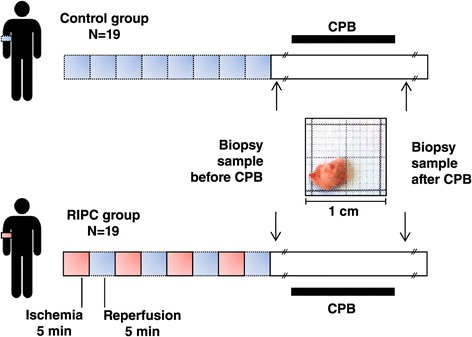
**Experimental setting.** RIPC was performed by 4 cycles of 5 minutes of upper arm ischemia induced with a blood pressure cuff which was inflated to 200 mmHg. Each cycle of ischemia was followed by 5 minutes of reperfusion. In the control group 4 cycles of 5-minutes blood-pressure cuff inflation to 20 mmHg and 5-minutes cuff deflation were applied. Cardiac biopsies were obtained before and after CPB. CPB, cardiopulmonary bypass; RIPC, remote ischemic preconditioning.

Aim of the study was to identify cell stress associated proteins in cardiac tissue samples of cardiosurgical patients with and without RIPC and patients included into the study were selected based on the criteria described earlier [26]. Each patient (age ≥ 18 years) gave written informed consent to participate in the study. All types of cardiac surgery in which cardiopulmonary bypass (CPB) was used were included. Patients were randomized to group RIPC or control in a double-blinded fashion. RIPC was induced by four cycles of upper limb ischemia (5-minutes blood-pressure cuff inflation to 200 mmHg and 5-minutes cuff deflation) after induction of total intravenous anaesthesia (propofol and sufentanil). In patients assigned to the control group we used four cycles of 5-minutes blood-pressure cuff inflation to 20 mmHg and 5-minutes cuff deflation without any limb ischemia (Figure 1).

Demographic data, information regarding the comorbidities, medications and ICU (intensive care unit) data of the patients employed in the clinical trial and experimental sub-studies (NCT00877305) have been presented recently [26,27]. Cardiac tissue of the right atrium was collected from RIPC and control patients before as well as after CPB. Serum concentrations of cardiac troponin T (cTnT) were employed as a marker for myocardial damage [35,36] and in all experiments patients in the RIPC group displayed significantly lower (P < 0.001) levels of cTnT compared to control patients (data not shown).

### Cell stress proteome profiler arrays

Proteome profiling was performed using commercially available cell stress proteome profiler arrays (R&D Systems, Minneapolis, USA; #ARY018) and the manufacturer’s protocol provided with the assay kit. Expression levels of 26 cell stress associated proteins were evaluated in cardiac tissue that was derived from cardiosurgical patients (control and RIPC) before as well as after CPB. Equal amounts of protein (15 μg) of each biopsy sample were pooled and applied to the respective array membrane. Densitometric analyses of the arrays were performed using the ImageJ 1.41o software (ImageJ, NIH, USA). Only proteins with signal intensities ≥10% of the internal reference control protein spot were further analysed.

### Westernblotting

Based on the results of the cell stress proteome profiler arrays, thioredoxin was chosen as a promising candidate for RIPC-mediated cardioprotection [32-34] and Westernblotting was performed with samples of each patient separately. Protein extraction from cardiac tissue biopsies was performed with RIPA buffer containing 150 mM sodium chloride, 1.0% NP-40, 0.1% sodium dodecyl sulfate (SDS), 1% sodium deoxycholate, 50 mM Tris-HCl (pH 7.6; all from Sigma-Aldrich, Hamburg, Germany). Protein concentrations were determined with a BCA Protein Assay Kit (Pierce Biotechnology, Rockford, USA). Samples were boiled for 5 minutes after addition of SDS polyacrylamide gel electrophoresis (PAGE) sample buffer (62.5 mM Tris-HCl, 2% SDS, 10% glycerol, 5% β-mercaptoethanol, all from Sigma-Aldrich). An equal amount of protein (50 μg) of each sample was separated by 10% SDS-PAGE and transferred onto a PVDF membrane (Amersham Pharmacia Biotech, Piscataway, USA). Membranes were then incubated in blocking solution (Starting Block T20; Fisher Scientific, Schwerte, Germany) for 1 hour at room temperature, followed by an overnight incubation with specific antibodies against thioredoxin-1 (Cell Signalling Technology, Danvers, USA; 1:1.000) or β-actin (Santa Cruz, Heidelberg, Germany; 1:1.000), which served as a loading control. After washing in TBS containing 0.05% Tween 20 (Sigma-Aldrich), the membranes were incubated for 1 hour with horseradish peroxidase conjugated pig anti-rabbit immunoglobulin G (Dako, Glostrup, Denmark; 1:10.000) referring to the manufacturer’s instructions. The final reaction was visualized using enhanced chemiluminescence (ECL-Detection Reagents, GE Healthcare, Munich, Germany), and the membrane was exposed to x-ray film. Images were taken and the intensities of the respective bands were densitometrically analysed with the software ImageJ (v1.41o, NIH). Protein expression levels of thioredoxin were relativized to the intensity of the respective actin band and depicted as arbitrary units (a.u.).

### Enzyme Linked Immunosorbent Assay (ELISA)

Human thioredoxin ELISAs (Biozol, Eching, Germany; #USC-SEA702HU) were performed using unpooled samples from each patient separately. Briefly, protein extraction from cardiac tissue biopsies was performed with RIPA buffer as described above. 50 μg of total protein from each patient were transferred into each well of a 96-well plate and all further steps were performed according to the manufacturer’s protocol. The amount of thioredoxin was relativized to the total protein within the respective sample. As suggested in the manufacturer’s protocol, hemolytic tissue lysates (HTLs) were excluded from further analyses (control before CPB, 9 HTLs; control after CPB, 3 HTLs; RIPC before CPB, 0 HTLs; control after CPB, 0 HTLs).

### Statistical analysis

Statistics were performed using the software GraphPad Prism version 5.01 for Windows. Each data set was tested for normality using the D’Agostino and Pearson omnibus test. Two way ANOVA in combination with Bonferroni post-tests were employed for analyses of the Westernblotting results, while due to missing data points (excluded HTLs) in the control group, ELISA data were evaluated using unpaired T-tests. Variables are expressed as mean ± SEM.

## Results

### Profiling of cell stress protein expression in cardiac tissue

**Figure 2:**
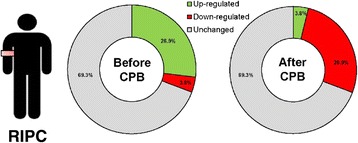
**Overall regulation of cell stress protein expression in the RIPC group (compared to the control group) before and after cardiopulmonary bypass (CPB).**

**Figure 3:**
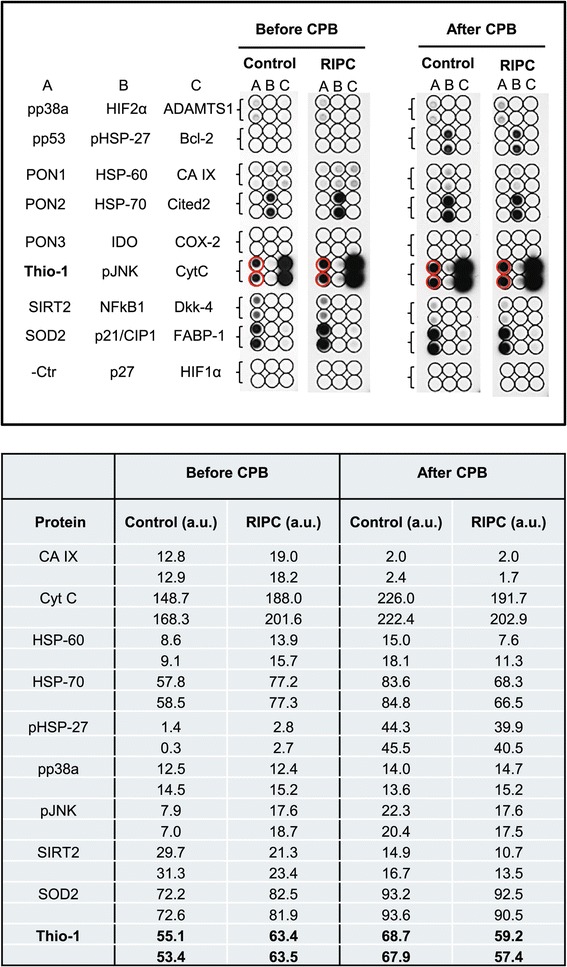
**Proteome profiling of cell stress proteins.** Equal amounts of protein from control (N = 18) and RIPC patients (N = 18) were pooled and employed in the array. Only proteins with signal intensities ≥10% of the internal reference control protein spot (not shown) were quantified. Numbers in the table represent the densitometric intensities of duplicate sample spots. pp38α, phospho-p38 alpha (T181/Y185); HIF2α, hypoxia inducible factor 2 alpha; ADAMTS1, a disintegrin and metalloproteinase with thrombospondin motifs 1; pp53, phospho-p53 (S46); pHSP-27, phospho heat shock protein-27; Bcl-2, B cell lymphoma-2; PON1, paraoxonase 1; HSP-60, heat shock protein-60; CA IX, carbonic anhydrase IX; PON2, paraoxonase 2; HSP-70, heat shock protein-70; Cited2, Cbp/p300-interacting transactivator; PON3, paraoxonase 3; IDO, indoleamine 2,3-dioxygenase; COX-2, cyclooxygenase-2; Thio-1, thioredoxin-1; pJNK, phospho c-Jun n-terminal kinase (T183/Y185); CytC, cytochrome C; SIRT2, sirtuin 2; NFkB1, nuclear factor kappa B1; Dkk-4, dickkopf-4; SOD2, superoxide dismutase 2; p21/CIP1, cyclin-dependent kinase inhibitor 1A; FABP-1, fatty acid binding protein-1; - Ctr, negative control; p27, cyclin-dependent kinase inhibitor 1B; HIF1α, hypoxia inducible factor 1 alpha; a.u., arbitrary units.

Proteome profiling employing cardiac tissue of cardiosurgical control patients and cardiosurgical patients undergoing RIPC revealed an increased expression of 26.9% (7/26) of stress associated proteins in RIPC tissue obtained before CPB [proteins up-regulated in the RIPC group: Carbonic anhydrase IX (CA IX), cytochrome C (Cyt C), heat shock protein-60 (HSP-60), heat shock protein-70 (HSP-70), phospho JNK (pJNK), superoxide dismutase 2 (SOD2), thioredoxin-1 (Thio-1)], while expression levels of 3.8% (1/26) of the proteins were down-regulated compared to the control group [proteins down-regulated in the RIPC group: Sirtuin 2 (SIRT2)]. In cardiac tissue obtained after CPB, only one of the proteins 3.8% (1/26) was slightly up-regulated in the RIPC group [proteins up-regulated in the RIPC group: Phospho-p38 alpha (pp38α)] while compared to the control group, expression levels of 26.9% (7/26) of the proteins were down-regulated [proteins down-regulated: Cytochrome C (Cyt C), heat shock protein-60 (HSP-60), heat shock protein-70 (HSP-70), phospho heat shock protein-27 (pHSP-27), phospho JNK (pJNK), sirtuin 2 (SIRT2), thioredoxin-1 (Thio-1); Figure 2]. A detailed analysis of the protein expression in control and RIPC cardiac tissue samples obtained before and after CPB is shown in Figure 3.

### Semi-quantitative evaluation of thioredoxin protein expression in cardiac tissue

**Figure 4:**
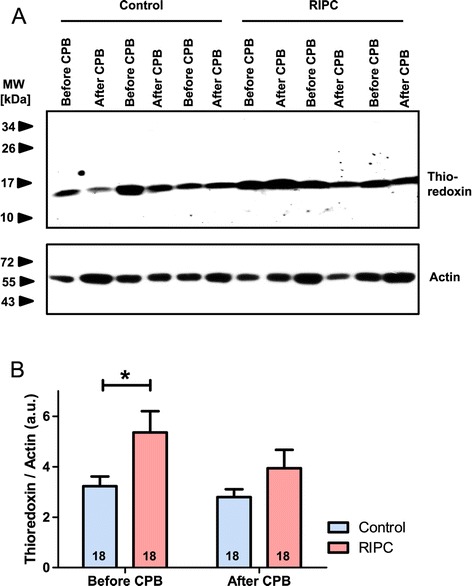
**Semi-quantitative evaluation of thioredoxin protein expression. A**: Representative Westernblotting experiment performed with cardiac tissue samples of 3 control and 3 RIPC patients. **B**: Evaluation of the relative protein expression levels of thioredoxin in control and RIPC patients. Numbers in the columns display the numbers of patients employed in the respective experiment. MW, molecular weight; kDa, kiloDalton; a.u., arbitrary units; columns display the mean; bars denote SEM; *, P < 0.05, two way ANOVA with Bonferroni post-test.

One of the proteins that showed a strong expression within the cardiac tissue and that was also regulated by RIPC is thioredoxin, a small redox protein and at least in animal studies, a possible candidate for cardioprotection [32,33,37,38]. Based on our results of the human proteome profiling approach which was performed with pooled tissue samples of the respective patients, we decided to further focus on thioredoxin as a potential molecule conferring RIPC-mediated cardioprotection and investigated the expression of thioredoxin in each patient separately. Westernblotting results mainly confirmed the outcome of the protein arrays, showing an increased expression of thioredoxin in cardiac tissue of RIPC patients that was obtained before CPB (RIPC: 5.36 ± 0.85 a.u.; control: 3.23 ± 0.39 a.u.; P < 0.05, Figure 4), while no statistically significant differences in thioredoxin protein expression levels were detected in samples taken after CPB (RIPC, 3.94 ± 0.73 a.u.; control, 2.79 ± 0.31 a.u.; P > 0.05; Figure 4).

### Quantification of thioredoxin protein expression in cardiac tissue

**Figure 5:**
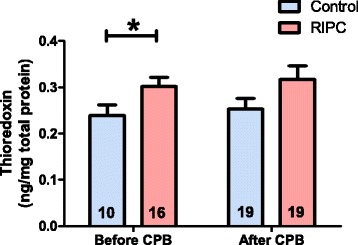
**Quantification of thioredoxin protein expression by ELISA.** The amount of thioredoxin protein is increased in tissue from RIPC patients that was obtained before CPB. Numbers in the columns display the numbers of patients employed in the respective experiment. Columns display the mean; bars denote SEM; *, P < 0.05, unpaired T-test.

Based on the results of the semi-quantitative Westernblotting experiments, we furthermore performed thioredoxin specific ELISAs to quantify the amount of the protein within the respective cardiac tissue samples of each patient and correlate it to the total amount of protein within the sample. A statistically significant increase of thioredoxin protein expression was again only detected in samples of RIPC patients that were obtained before CPB (RIPC: 0.30 ± 0.02 ng/mg protein; control: 0.24 ± 0.02 ng/mg protein; P < 0.05; Figure 5), while a tendency towards increased thioredoxin expression was found in the RIPC tissue samples taken after CPB (RIPC: 0.32 ± 0.03 ng/mg protein; control: 0.25 ± 0.02 ng/mg protein; P = 0.05; Figure 5).

## Discussion

Several clinical trials have suggested RIPC as an efficient and straightforward intervention to protect the heart from ischemia reperfusion injury which is a common perioperative complication during various surgical procedures [8,9,13,16,39-41]. Despite these encouraging clinical data on RIPC-mediated cardioprotection, the underlying mechanisms -especially within the myocardial tissue- are still discussed controversially [17,20]. Detailed knowledge about the molecular and cellular events that are associated with RIPC-mediated cardioprotection could however help to increase the effectiveness of RIPC and further improve patient outcome. Therefore, in the last years we have conducted several studies and investigated the effects of RIPC on ischemia/reperfusion injury in the heart focussing on the potential mechanisms that transfer the RIPC signal within the myocardium [26,27,42,43].

In the study presented, we performed proteomic profiling using cardiac tissue from cardiosurgical patients with cardiopulmonary bypass (CPB) that were subjected to RIPC or sham intervention.

Several stress associated proteins were found to be strongly expressed in tissue obtained after CPB. In myocardial samples of the control group, heat shock proteins (HSP) and especially the phosphorylated form of HSP-27 are up-regulated after CPB. These findings are not surprising as surgery as well as the implementation of the CPB are stress factors and other groups have also shown that cardiac surgery results in an increased expression of heat shock proteins within the respective tissue [44,45].

However, protein profiling also revealed that in tissue obtained before CPB, several of the cell stress associated protein [e.g. HSP-70, cytochrome C (CytC), superoxide dismutase 2 (SOD2), thioredoxin-1 (Thio-1)] are up-regulated in the RIPC group. This finding is of major interest as an increased expression of cell stress proteins may have a positive effect on the heart [46] and can induce cardioprotective mechanisms [47-49]. Animal studies suggested that besides the heat shock proteins, redox proteins like thioredoxin may also play a role in cardioprotection [32,33,50,51]. Thioredoxin is a small oxidoreductase enzyme which is ubiquitously found in many organisms from plants and bacteria to mammals. It represents a potent redox protein acting as antioxidant by facilitating the reduction of other proteins by cysteine thiol-disulfide exchange thereby abolishing oxidative stress within cells and tissues [28,29]. Concerning the protein expression of thioredoxin within the cardiac tissue, our cell stress proteome profiler arrays, Westernblotting experiments and thioredoxin ELISAs all revealed that RIPC leads to a statistically significant increased expression of the protein in cardiac tissue obtained before, but not after CPB. These findings are in accordance with our recently published studies, which also suggested that RIPC-mediated effects are mainly evident in tissue derived before but not in tissue obtained after CPB [26,27]. The reasons for this observation might be based on the fact that the immunological response to the extracorporeal circulation during CPB generates a systemic inflammatory response syndrome (SIRS) that is associated with the release of various cytokines [52,53] which may mask possible RIPC-mediated effects in tissue obtained after CPB. On the other hand, it is known that ischemic preconditioning represents a biphasic phenomenon with a first and a second window of protection [54]. The early phase of protection develops quickly within minutes from the initial ischemic conditioning event and lasts for 2 to 3 hours. This is followed by a delayed phase that begins after 12 to 24 hours and lasts up to 4 days. In our study, samples taken before CPB fall into the first window of protection, while tissue that is derived after CPB possibly falls into the gap between the two windows, which explains why only few proteins are found to be up-regulated by RIPC in tissue samples derived after CPB.

Our findings that several cell stress associated proteins, including thioredoxin, exhibit a temporary upregulation by RIPC may be of value for future studies employing RIPC to reduce morbidity and mortality in cardiosurgical patients. In a clinical perspective, cardiosurgical patients seem to profit from an early and temporary induction of cell stress associated proteins by transient episodes of remote ischemia before surgery. However, the RIPC-induced response in the remote target organ (e.g. heart) most likely relies on a delicate balance between protective and potentially harmful RIPC mediators - circulating factors as well as local target tissue derived molecules. The expression of these mediators needs to be precisely adjusted to exploit the cardioprotective potential of RIPC and to avoid possible adverse effects of RIPC. Therefore, optimizing the currently available RIPC protocols [8,55] by adjusting the RIPC location, timing, duration and cycle numbers as well as taking into consideration possible comorbidities of RIPC patients [56] may enhance the potential of RIPC-mediated cardioprotection, resulting in an improved patient outcome and may help to reduce morbidity as well as mortality after cardiosurgical interventions.

There are some limitations of the study which need to be considered: (i) Post-operative or clinical endpoints were not evaluated in the experimental study presented. However, clinical outcome parameters are described in the context of our previous pilot study (Additional file 1: Table S1; [42,43]). (ii) Depending on the patient and surgical procedure the first biopsy (before CPB) was collected between 15 minutes and 100 minutes after RIPC. As the second biopsy sample was collected immediately after CPB the time period between the collection of the first and second sample varied between the patients. (iii) Based on surgical and ethical limitations only cardiac tissue of the right atrium and no ventricular tissue could be obtained and we cannot exclude the possibility that tissue taken after CPB was affected by e.g. shear stress induced by the suture of the venous cannula (iv) Due to methodological constraints and the limited amount of cardiac tissue available, not all experiments could be performed with samples of all patients. However, it was assured that in all experiments patients in the RIPC group had reduced myocardial damage, which was demonstrated by significantly lower serum cTnT levels in the RIPC group.

## Conclusions

In the present study we performed proteomic profiling with heart tissue obtained from cardiosurgical patients that were subjected to RIPC or sham intervention. We provide evidence for thioredoxin as a RIPC-induced factor in the heart of cardiosurgical patients and identified several cell stress associated proteins that are regulated by RIPC and may play a role in RIPC-mediated cardioprotection.

## Additional file

Additional file 1: Table S1. Clinical and experimental trials with relation to the actual study.
