# Supplementary figures and images for: Profiling of cell stress protein expression in cardiac tissue of cardiosurgical patients undergoing remote ischemic preconditioning: implications for thioredoxin in cardioprotection
Source: J Transl Med. 2015 Jan 27;13:34. doi: 10.1186/s12967-015-0403-6 (PMC4316390; doi:10.1186/s12967-015-0403-6)

## Slide 1
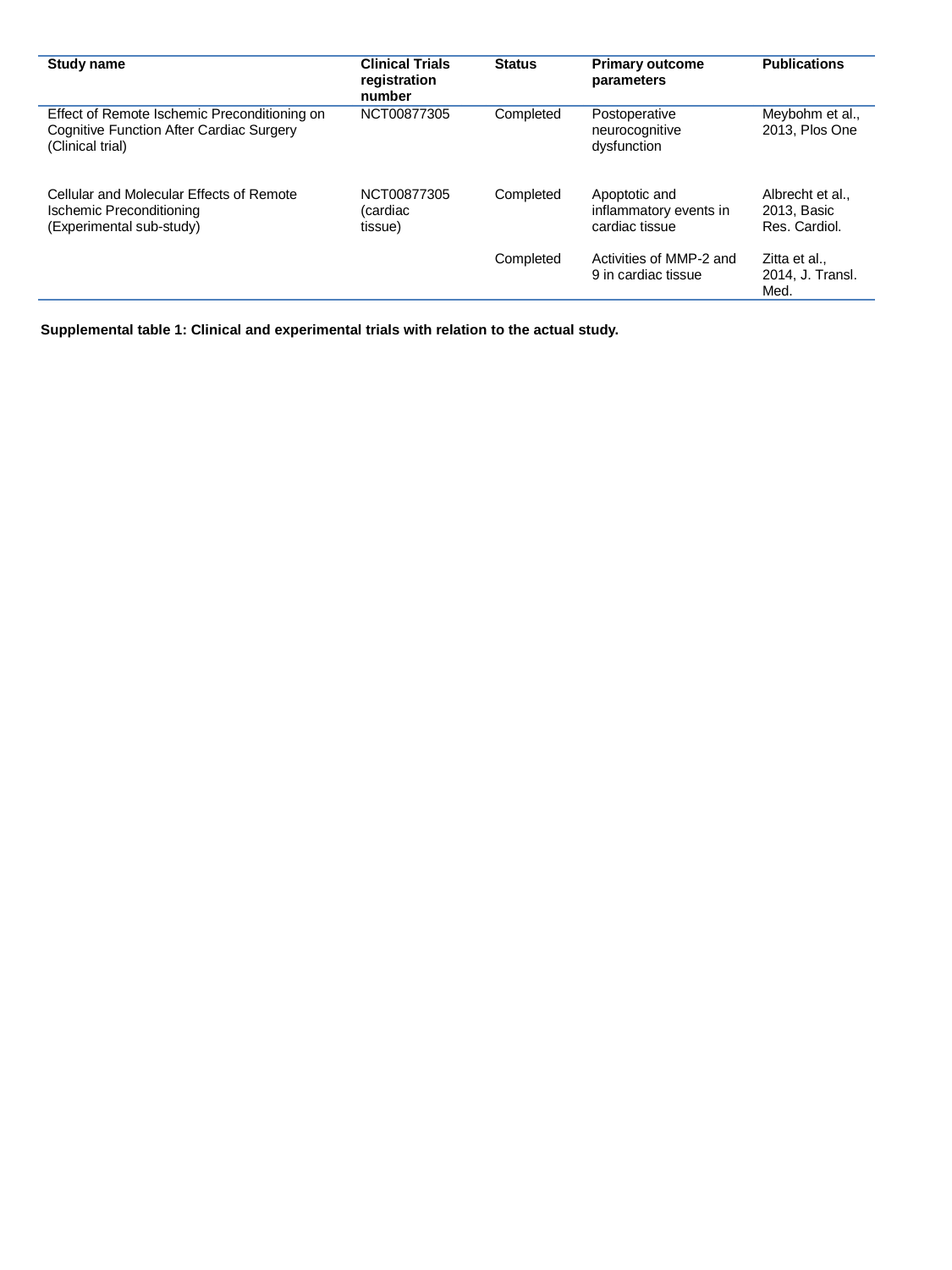

Supplemental table 1: Clinical and experimental trials with relation to the actual study.

Supplement: Additional file 1: Table S1. — Clinical and experimental trials with relation to the actual study. [file 12967_2015_403_MOESM1_ESM.pptx]
